# Sex differences in the predictability of risk-taking behavior

**DOI:** 10.1093/beheco/arac105

**Published:** 2022-12-13

**Authors:** Jack A Brand, Jason Henry, Gabriela C Melo, Donald Wlodkowic, Bob B M Wong, Jake M Martin

**Affiliations:** School of Biological Sciences, Monash University, Melbourne, Victoria 3800, Australia; School of Science, RMIT University, Melbourne, Victoria 3083, Australia; School of Biological Sciences, Monash University, Melbourne, Victoria 3800, Australia; School of Science, RMIT University, Melbourne, Victoria 3083, Australia; School of Biological Sciences, Monash University, Melbourne, Victoria 3800, Australia; School of Biological Sciences, Monash University, Melbourne, Victoria 3800, Australia; Department of Wildlife, Fish, and Environmental Studies, Swedish University of Agricultural Sciences, Umeå SE-901 83, Sweden

**Keywords:** behavioral consistency, behavioral syndrome, behavioral type, personality, reaction norm

## Abstract

Recent research has found that individuals often vary in how consistently they express their behavior over time (i.e., behavioral predictability) and suggested that these individual differences may be heritable. However, little is known about the intrinsic factors that drive variation in the predictability of behavior. Indeed, whether variation in behavioral predictability is sex-specific is not clear. This is important, as behavioral predictability has been associated with vulnerability to predation, suggesting that the predictability of behavioral traits may have key fitness implications. We investigated whether male and female eastern mosquitofish (*Gambusia holbrooki*) differed in the predictability of their risk-taking behavior. Specifically, over a total of 954 behavioral trials, we repeatedly measured risk-taking behavior with three commonly used assays—refuge-use, thigmotaxis, and foraging latency. We predicted that there would be consistent sex differences in both mean-level risk-taking behavior and behavioral predictability across the assays. We found that risk-taking behavior was repeatable within each assay, and that some individuals were consistently bolder than others across all three assays. There were also consistent sex differences in mean-level risk-taking behavior, with males being bolder across all three assays compared to females. In contrast, both the magnitude and direction of sex differences in behavioral predictability were assay-specific. Taken together, these results highlight that behavioral predictability may be independent from underlying mean-level behavioral traits and suggest that males and females may differentially adjust the consistency of their risk-taking behavior in response to subtle changes in environmental conditions.

## Teaser

Individuals often differ in how consistently they express their behavior (i.e., behavioural predictability). Recent research suggests that variation behavioral predictability is heritable and has key fitness consequences. We showed that male and female eastern mosquitofish (*Gambusia holbrooki*) differed in the predictability of their risk-taking behavior. Furthermore, we found that both the direction and magnitude of these sex differences were context specific.

## INTRODUCTION

There is increasing evidence that within populations, individuals consistently differ from one another in both their mean-level behavioral traits (i.e., animal personality; Sih et al. 2004; Bell et al. 2009) as well as how they alter their behavior over time or in response to environmental change (i.e., behavioral plasticity; Dingemanse et al. 2010; Dingemanse and Wolf 2013). As behavior enables animals to interact with and adapt to their environment, it is not surprising that these individual differences in behavior have major implications for species ecology. Indeed, previous research has suggested that animal personality and plasticity can influence population persistence and stability (Réale et al. 2007; Sih et al. 2012; Wolf and Weissing 2012; Dingemanse and Wolf 2013). Further, these discrete behavioral differences between individuals are often heritable (Dingemanse et al. 2002; Dochtermann et al. 2015; Araya-Ajoy and Dingemanse 2017), consistent across multiple ecological contexts (i.e., behavioral syndromes; Sih et al. 2004) and may affect organismal fitness (Moirón et al. 2019; Munson et al. 2020), suggesting that individual differences in behavior are likely to have substantial evolutionary consequences.

**Figure 1:**
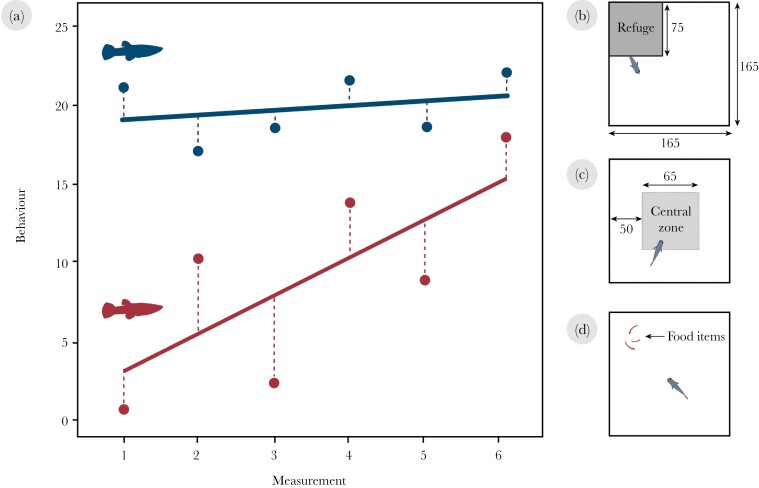
(a) Conceptual illustration of individual differences in personality, plasticity, and predictability adapted from Jolles et al. (2019). The hypothetical plot shows a red and blue individual repeatedly measured for their behavior over six consecutive measurements. Hypothetical linear regression fits to the individual data are shown. The two individuals differ in their personality (intercept of the two lines), plasticity (slope of the two lines), and predictability (residuals; dashed lines). Schematic diagrams of (b) refuge-use, (c) thigmotaxis, and (d) foraging assays where the time spent outside of the refuge, the time spent in the central zone, and latency to forage, respectively, were quantified for each individual fish as three separate measures of risk-taking behavior. All measurements are in mm. Fish not to scale.

Furthermore, research has now shown that individuals may also differ in their residual intra-individual behavioral variation (i.e., behavioral predictability or rIIV; Stamps et al. 2012; Biro and Adriaenssens 2013; Westneat et al. 2015; Mitchell et al. 2021; Figure 1a). This variation in behavioral predictability is found even after accounting for individual differences in both personality and plasticity (Cleasby et al. 2015; Jolles et al. 2019; Hertel et al. 2021). For example, analysis of daily movement distances in wild female brown bears (*Ursus arctos*) found that some individuals were more predictable than others in their activity (Hertel et al. 2021). More specifically, there was a five-fold (from 1.1 to 5.5 km) difference across individuals in the standard deviation of their daily movement distance (Hertel et al. 2021). Despite the majority of reported behavioral variation occurring within-individuals (Bell et al. 2009), these individual differences in predictability are still often overlooked or ascribed to random statistical noise (Westneat et al. 2015). However, recent work has found that variation in behavioral predictability may be underpinned by heritable genetic variance (Martin et al. 2017; Prentice et al. 2020) and have survival consequences (Jones et al. 2011; Chang et al. 2017), suggesting that behavioral predictability may constitute an evolvable trait with substantial implications for organismal fitness.

While the ecological and evolutionary consequences of behavioral predictability are still not clear, recent work has suggested that decreased behavioral predictability may be an adaptive strategy for dealing with risky situations (Briffa 2013; Chang et al. 2017; but see Urszán et al. 2018). Indeed, research investigating predator-prey dynamics in jumping spiders (*Portia labiata* and *Cosmophasis umbratica*) found that the predictability of prey species’ behavior was key to their survival when faced with a predator (Chang et al. 2017). Similar research using computer simulations reported that human participants were less likely to capture more unpredictable “prey” in a computer game, again suggesting that decreased predictability may aid in predator avoidance (Jones et al. 2011). Furthermore, recent work found that both common hermit crabs (*Pagurus bernhardus*) and common pill bugs (*Armadillidium vulgare*) were more unpredictable in their risk-taking behavior when in more risky, unfamiliar environments (Briffa 2013; Horváth et al. 2019). Taken together, this research suggests that decreased behavioral predictability may be an adaptive strategy for dealing with predation risk in precarious environments.

Despite the potential fitness implications of behavioral predictability, little is known about the intrinsic factors which may drive differences in intra-individual behavioral variation. In particular, it is possible that variation in behavioral predictability may differ between males and females. Given the associations between predictability and predation risk (Jones et al. 2011; Briffa 2013; Chang et al. 2017), this may be especially true for species in which males and females differ in their vulnerability to predation. However, research on sex differences in predictability is limited and the studies that do exist report mixed results. For example, while a previous study reported that female guppies (*Poecilia reticulata*) were less predictable than males in their lateralization (McLean and Morrell 2020), other research has found no sex differences in the predictability of risk-taking behaviors (White and Briffa 2017; Horváth et al. 2019; Prentice et al. 2020). It remains unknown, however, whether these patterns are found more broadly across species and multiple behavioral traits. As behavioral predictability has been shown to be a heritable trait (Martin et al. 2017; Prentice et al. 2020) with important implications for organismal survival (Chang et al. 2017), understanding how males and females differ in their behavioral predictability across a range of behavioral traits is key to understanding the potential ecological and evolutionary consequences of intra-individual behavioral variation.

Accordingly, we investigated sex differences in behavioral predictability in the eastern mosquitofish (*Gambusia holbrooki*). The eastern mosquitofish is a small, sexually dimorphic poecilid fish native to the United States of America that has been introduced into freshwater ecosystems around the world (Pyke 2008). Due to their small body size, mosquitofish are often a target of predation by wading birds, larger fish, decapod crustaceans, and predatory insect larvae (Britton and Moser 1982; Horth 2004; Pyke 2008). The species is also highly social, often forming large, mixed-sex schools (Pyke 2005). However, female *Gambusia* sp. tend to be more social and less bold than male conspecifics (Heinen-Kay et al. 2016; Lagesson et al. 2019; Martin et al. 2019a; Michelangeli et al. 2020). Since male mosquitofish are sexually aggressive (Pilastro et al. 2003), and larger female *Gambusia* sp. are often more vulnerable to predation than their smaller male counterparts (Britton and Moser 1982), this increased sociability and decreased boldness in females likely represents an adaptive strategy for dealing with increased predation risk and/or to escape costly male harassment. Indeed, previous research has shown that predation pressure has strong effects on boldness in poecilid fish, particularly in females (Harris et al. 2010; Heinen-Kay et al. 2016). Together, these findings suggest that male and female mosquitofish are likely exposed to differing levels of predation risk, resulting in sex differences in risk-taking behaviors. Given the previously reported associations between behavioral predictability and risk-management (Briffa 2013; Horváth et al. 2019), these sex differences in boldness and predation risk make mosquitofish an ideal model species to investigate potential sex differences in the predictability of risk-taking behavior.

We, therefore, repeatedly measured risk-taking behavior in both male and female mosquitofish. We used three separate tests of risk-taking behavior—refuge-use, thigmotaxis, and foraging latency—to ensure that any sex differences in behavioral predictability were general patterns that were robust to assay design. Due to the increased vulnerability to predation and decreased boldness often found in female *Gambusia* sp. (Britton and Moser 1982; Heinen-Kay et al. 2016; Martin et al. 2019a; Michelangeli et al. 2020), we predicted that female mosquitofish in the current study would be less bold than their male counterparts across all three behavioral assays. However, while we expected that decreased behavioral predictability may form part of a general risk-management strategy and would thus vary between the sexes, the direction of this effect was more difficult to predict. For example, we may expect females to be less predictable than males due to their larger body size and subsequent vulnerability to predation. Conversely, males are often bolder than females (see above), and thus may be less predictable in order to offset the potential increase in predation risk due to their riskier behavior. Therefore, while we predicted that sexes would consistently differ in their behavioral predictability across all three assays, we had no clear expectations about the direction of this effect.

## METHODS

### Study species and animal husbandry

Wild-caught eastern mosquitofish (*n* = 48) of equal sex-ratio were collected using seine nets from the Science Centre Lake at Monash University, Australia (37°54ʹ28″S, 145°08ʹ16″E) in October 2020. Individuals were sexed based on the presence/absence of a gonopodium (male reproductive organ) and a dark peritoneal spot (present in mature females; Pyke 2005). Fish were transported to animal facilities at Monash University where they were housed in same-sex tanks (600 × 300 × 300 mm; 24 fish per tank) filled with aged, carbon-filtered freshwater. Housing tanks were located within a controlled-temperature room (12:12 h light:dark cycle) maintained at 19 ± 0.5 °C. Each tank was provided with a biofilter, 20 mm of fine pebble substrate and contained plastic plants for shelter. Animals were acclimated to laboratory conditions for one week prior to the start of experiments. All fish were fed a combination of commercial fish food (Otohime Hirame larval diet; 580–910 µm) and chironomid larvae ad libitum 5 out of 7 days of the week, while 50% water changes were performed weekly.

Twenty-four hours before behavioral experiments, all fish were sorted into individual tanks (165 × 165 mm) where they remained housed for the duration of the experimental period. Tanks contained a small, opaque refuge (75 × 75 mm) located above the water surface in one corner for shelter, and each tank was placed into one of three water baths (water depth = 70 mm) maintained at 18.94 ± 0.28 °C (mean ± SD) within a controlled-temperature room. Each water bath contained 16 individual tanks with an equal mix of males and females (i.e., 8 males and 8 females placed in individual tanks within a single water bath). Individual tanks were made from opaque white plastic to optimize automated tracking and to visually isolate fish from conspecifics to avoid the influence of visual social cues during trials. However, tanks also contained 24 small (1 mm) perforations around the walls that allowed water flow between the tank and the surrounding water bath. This was done to maintain high water quality within individual tanks. Testing fish within these modified housing tanks also served to reduce handling and transport stress during the experiment. The feeding and water changing schedule during individual housing were the same as that described above (see Supplementary Table S1).

### Behavioral experiments

All fish were tested for risk-taking behavior across three separate assays. In brief, fish underwent 1) a refuge-use assay, where we recorded the total time spent outside of a refuge, 2) a thigmotaxis assay testing wall-hugging as a separate measure of risk-taking behavior, and 3) a foraging assay where we recorded the latency to forage as a measure of each individual’s willingness to feed in a potentially risky environment. All three assays have previously been used to measure risk-taking behavior in fish (Magnhagen and Borcherding 2008; Kotrschal et al. 2014; Houslay et al. 2018; Jolles et al. 2019). Both the refuge and thigmotaxis assays were carried out twice daily on alternating days (Figure 1b, c). Specifically, behavioral trials were carried out once in the morning (09:00–11:00) and once in the afternoon (14:00–16:00), with trial types alternating everyday (i.e., day one = 2 × refuge trials; day two = 2 × thigmotaxis trials etc.), except for foraging trials which were only carried out in the afternoons (16:00–17:00). For each fish, this process was repeated over 10 experimental days with a 2-day break period in the middle. This provided eight repeated measurements for refuge-use and thigmotaxis assays for each fish (see Supplementary material). Foraging trials were repeated five times over the course of the experiment. All behavioral experiments were carried out in individual home tanks with trials video-recorded using a HC-V180 camcorder (Panasonic) mounted directly above the tanks. Animal tracking and behavioral analysis was conducted using the Ethovision XT v.15 software (Noldus Information Technologies, Wageningen, the Netherlands) for refuge-use and thigmotaxis assays, as well as the event-logging software BORIS (Friard and Gamba 2016) for foraging trials (see Supplementary material).

### Refuge-use assay

All fish were tested for refuge-use as a measure of risk-taking behavior in their individual tanks (Figure 1b). Briefly, an opaque, square shelter (75 × 75 mm) was located above the water surface in one corner of the tank. This created a sheltered region within the tank in which the fish could hide. We recorded the total time that each individual spent out of the refuge (i.e., in the open) as a measure of risk-taking behavior over the following 15 min trial. Here, higher scores represent more risk-prone individuals. After a minimum of three hours, trials were repeated in the afternoon. In 29 of the 333 trials (~ 8.7 % of the data), fish never left the refuge. However, these trials were maintained in all analyses as they represent biologically meaningful variation in risk-taking behavior.

### Thigmotaxis assay

Fish were also tested for risk-taking behavior in a thigmotaxis assay (Figure 1c). Shelters were removed from each tank prior to the start of the trial. Each individual tank contained an exposed “central zone” (65 × 65 mm) located in the center of the tank, 50 mm from each wall. Thigmotaxis (i.e., wall preference behavior) is a common fear response in fish (Kotrschal et al. 2014; Polverino et al. 2019). Similar to Kotrschal et al. (2014), we therefore recorded the total time spent in the “central zone” as a measure of risk-taking behavior over the course of the 15 min trial. Here, higher scores represent more risk-prone individuals. Similar to the refuge-use assay, trials were repeated again in the afternoon after a minimum of three hours. In 33 of the 382 trials (~ 8.6 % of the data), fish never entered the exposed central zone. However, again these trials were maintained in all analyses as they represent biologically meaningful variation in risk-taking behavior.

### Foraging assay

All fish were tested for their latency to forage as a separate measure of risk-taking behavior (Figure 1d). Protocols were adapted from previously established methods in freshwater fish (Martin et al. 2019b; Brand et al. 2021b). All fish were fasted for approximately 24–48 h prior to foraging trials (see Supplementary Table S1). Further, all foraging assays were performed simultaneously to standardize hunger levels between individuals. Briefly, an opaque white plastic cylinder (75 mm diameter) was placed into the top corner of each individual’s tank. Three chironomid larvae (i.e., food items) were placed within the cylinder, with fish allowed to acclimate to testing conditions for 5 min. After acclimation, cylinders were removed, and fish behavior was recorded for 15 min. We recorded the latency to first consume a food item (hereafter foraging latency). Fish that did not consume a food item during the trial were given a score of 900 s (i.e., total duration of the assay; total number of occurrences = 5/239 trials).

Following the completion of experiments, fish were blotted dry with a paper towel and weighed to the nearest 0.0001 g using an analytical balance (Scientech ZSA210, Melbourne, Australia).

### Statistical analysis

Data were analyzed using *R* version 4.0.3 (R Core Team 2019). One round of refuge-use trials had to be discarded due to issues with video recordings. This resulted in a total of 954 behavioral trials (totaling 238.5 h) from 48 fish included in the analysis. Mass data were also lost for one individual. We therefore estimated mass data from sex-specific means to maintain this individual in the analysis. However, we also ran supplementary models analogous to those described below where the individual with mass estimated from sex-specific means was excluded from analyses to ensure that any sex differences in behavior were robust to the inclusion of this individual. The results from these models were qualitatively similar to those reported in the main text (see Supplementary Table S2). Time spent out of the refuge and time in the central zone were both square-root transformed, while foraging latency was log_10_ transformed to approximate a Gaussian error distribution. Further, we inverted foraging latency scores (i.e., foraging latency × −1) so that higher scores represented bolder individuals. Trial number (1–8) was defined so that the first trial = 0, while all response variables, continuous covariates (i.e., mass), and time of day were mean-centered (mean = 0, SD = 1: am = −0.5; pm = 0.5) prior to analysis to aid in model fitting and interpretation of parameter estimates.

We first used a Bayesian multivariate generalized linear mixed-effects model (*brms* package; Bürkner 2017) using all the repeated measures data to investigate whether individuals were consistent in their mean-level risk-taking behavior across all three assays (see Supplementary material; Table S3 for model output). More specifically, this model was used to estimate behavioral repeatability, as well as among-individual behavioral correlations between each measure of risk-taking behavior from the three assays. In our model, we fitted time spent out of the refuge, time in the central zone, and foraging latency as three separate dependent variables. Mass, time of day (am vs pm), trial number (1–8), sex, and a sex by trial number interaction were included as fixed-effects. However, time of day was not included in the foraging latency model as foraging trials were only performed in the afternoon. Sex was centered (i.e., female = −0.5; male = 0.5) in the multivariate model in order to provide variance estimates for the “average” fish, regardless of sex (Hertel et al. 2021). Preliminary analysis found no significant effect of the water bath (1–3) that fish were housed in on behavior and, therefore, was not included as a covariate in the final model to reduce model complexity. Individual identity (1–48) and trial number were included as random intercepts and slopes, respectively. We allowed correlations to vary among individuals to estimate whether fish were consistent in their risk-taking behavior across the three assays. This multivariate model was also used to calculate the repeatability of each measure of risk-taking behavior. Repeatability represents the proportion of total behavioral variance that is due to differences among individuals. However, as there was variation among individuals in their behavior over time (i.e., random slopes) as well as correlations between average behavior and behavioral plasticity (i.e., intercept–slope correlations), the proportion of behavioral variation that is due to among individual differences changes as a function of trial number. We therefore followed methods described in Briffa et al. (2013) to calculate short-term conditional repeatability (*R*_cond_; Equation 1) as:


$$R_{\text{cond}}=\frac{\left(V_{\text{int}}+2\times {\text{COV}}_{\text{is}}\times X+V_{\text{slope}}\times X^{2}\right)}{\left[\left(V_{\mathrm{int}}+2\times {\text{COV}}_{\text{is}}\times X+V_{\text{slope}}\times X^{2}\right)+V_{\text{residual}}\right]}$$ (1)

where $V_{\text{int}}$ represents the variance among intercepts, $V_{\text{slope}}$ is the variance among slopes, $\;V_{\text{residual}}$ represents the residual variance, ${\text{COV}}_{\text{is}}$ is the covariance between intercepts and slopes, and X represents trial number in our case. Here, we report *R*_cond_ at the intercept (i.e., where trial number = 0; see Supplementary Table S4 for trial specific repeatability estimates).

We then fitted three separate Bayesian, hierarchical linear mixed-effects models to investigate sex differences in mean- and residual-level risk-taking behavior. Hierarchical generalized linear mixed-effects models and their mathematical notation have been described in detail elsewhere (Cleasby et al. 2015). Briefly, these models allow the fitting of fixed and random-effects to both a “mean” and “residual” model. While the “mean model” allows the estimation of mean-level behavioral types, the “residual model” allows the estimation of residual intra-individual variation (i.e., behavioral predictability or rIIV) around that behavioral type (Hertel et al. 2021). In these models, we included time spent out of the refuge, time in the central zone, and foraging latency as three separate dependent variables. The mean models contained mass, time of day (for refuge and central zone use models), sex, and trial number as fixed-effects. Further, we included a sex by trial number interaction in the model to investigate whether males and females differed in how they altered their risk-taking behavior over repeated trials. Again, we included individual ID and trial number as random intercepts and slopes, respectively. We also fitted sex as a fixed effect factor in the residual model to investigate how sexes differed in the predictability of their risk-taking behavior within each assay context.

Similar to Brand et al. (2021a), all models were run on four chains using relatively uninformative, default priors for a total of 5000 iterations, with a warm-up of 1000, and a thinning interval of 2. Model convergence was verified with sufficient mixing of trace plots, with all $\hat{R}$ values = 1. We report posterior means with 95% credible intervals (CrI), with inference based on non-overlapping CrI’s with zero (i.e., clear evidence for an effect was considered when CrI’s did not include zero).

### Ethical note

Research was conducted in accordance with relevant Australian ethical guidelines and legislation, and all experimental procedures were approved by the Biological Sciences Animal Ethics Committee at Monash University (protocol number: 23461).

## RESULTS

### Individual-level effects: repeatability and across context correlations

Inspection of the random-effects in the multivariate model suggests that there were among-individual differences in risk-taking behavior across all three assays (i.e., personality). Indeed, estimates of short-term conditional repeatability at the intercept revealed that time out of the refuge (*R* [95% CrI] = 0.655 [0.536, 0.771]), time in the central zone (*R* [95% CrI] = 0.748 [0.661, 0.834]), and foraging latency (*R* [95% CrI] = 0.523 [0.331, 0.705]) were all repeatable measures of risk-taking behavior. Moreover, we found correlations among individuals in their behavior across all three assays (i.e., correlations among intercepts), suggesting that individuals were largely consistent in their mean-level behavior and that all three assays provide a valid measure of risk-taking behavior. More specifically, fish that spent more time out of the refuge also spent more time in the central zone (*r* [95% CrI] = 0.765 [0.562, 0.903]) and displayed a decreased foraging latency (*r* [95 % CrI] = 0.494 [0.175, 0.750]). Further, individuals that displayed a decreased foraging latency also spent more time in the central zone (*r* [95% CrI] = 0.411 [0.090, 0.676]). We also found moderate evidence for individual differences in behavioral plasticity (Supplementary Table S3), as well as correlations between individuals in their plasticity across the assays (i.e., plasticity syndromes; see Supplementary Tables S3 and S5 for intercept–slope, and slope–slope correlations amongst risk-taking behavior in each assay).

### Population-level effects on average behavior

**Figure 2:**
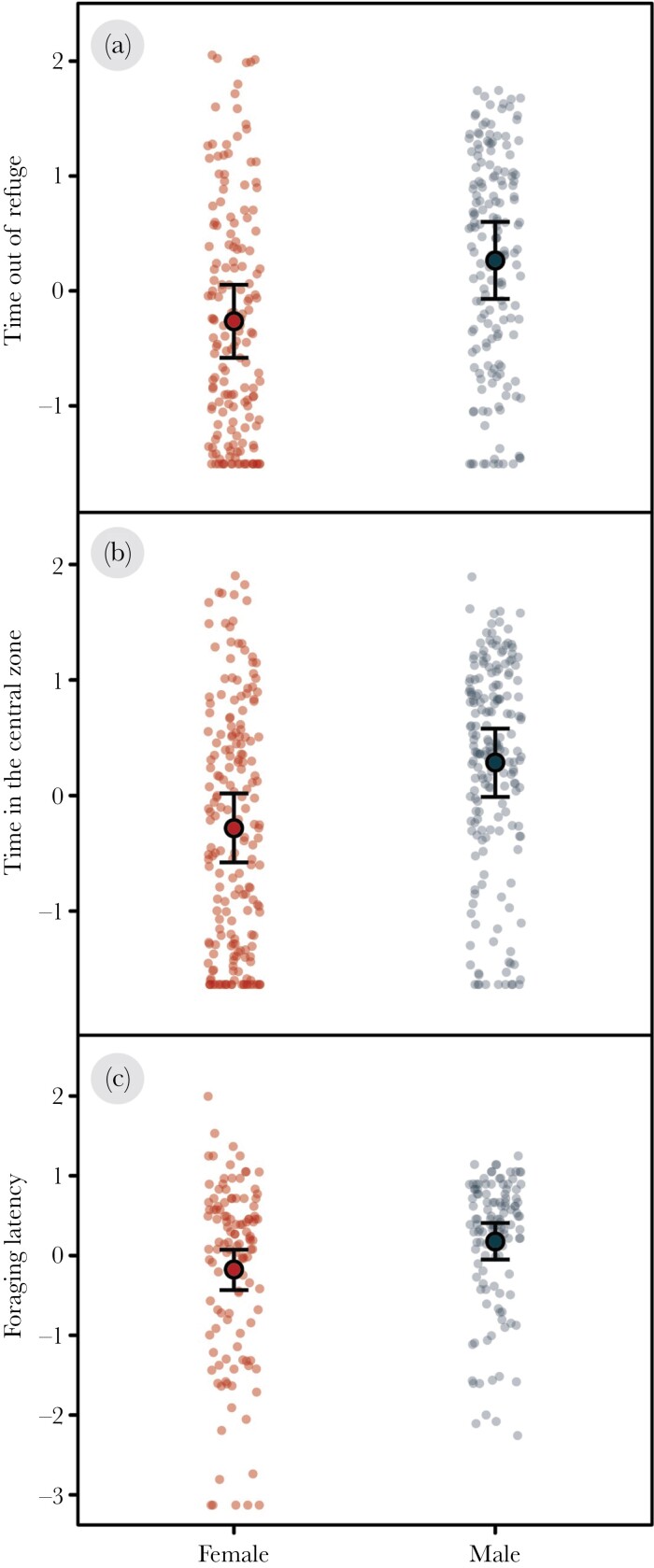
Sex differences in risk-taking behavior. Plots represent conditional effects (± 95% CrI) extracted from the Bayesian, hierarchical linear mixed-effects models for (a) time out of the refuge, (b) time in the central zone, and (c) foraging latency. Estimates are shown for both females (red) and males (blue). Red and blue semi-transparent points represent individual data points for females and males, respectively. Foraging latency scores were inverted so that higher scores represent bolder individuals that were quicker to feed. Behavioral scores are presented in standardized units.

On the population-level, we observed a similar pattern of results for risk-taking behavior across all three assays (Table 1). More specifically, male fish were bolder than females across all three assays. In particular, male fish spent more time out of the refuge (mean ± SD; male = 283 ± 219 s; female = 178 ± 222 s) and more time in the central zone (male = 214 ± 141 s; female = 138 ± 152 s), compared to their female counterparts (Table 1; Figure 2). Furthermore, in comparison to female conspecifics, male fish demonstrated moderately decreased foraging latencies (male = 19 ± 41 s; female = 69 ± 190 s; Table 1; Figure 2). However, this sex difference in foraging latency was only partially supported, with credible intervals overlapping with zero (Table 1). We also found a moderate effect of mass on foraging latency, with larger fish being marginally quicker to feed than their smaller counterparts (Table 1). However, again, there was some uncertainty around this estimate with credible intervals including zero. There was no substantial evidence for an effect of mass on time out of the refuge or time in the central zone (Table 1). Further, fish became consistently bolder over time in all three assays, with individuals increasing their time spent out of the refuge and their time spent in the central zone as well as displaying a decrease in foraging latency over repeated trials (Table 1). In contrast, however, time-of-day effects were only observed during the refuge-use assay, with fish decreasing the time they spent out of the refuge in the afternoons compared to the morning trials (Table 1). Finally, there was no evidence of an interaction between sex and trial number on risk-taking behavior in any of the three assays (Table 1).

**Table 1:** Model output from Bayesian, hierarchical generalized linear mixed-effects models. Estimates with 95% credible intervals are presented for each separate measure of risk-taking behavior. Foraging latency scores were inverted so that higher scores represent more risk-prone individuals that were quicker to feed. Bold text indicates fixed-effects estimates and intercept–slope correlations which were different from zero. Random-effects are presented in standard deviation (sd) units and the residual model is presented on the log scale

	Time out of refuge	Time in central zone	Foraging latency (inverted)
**Mean-model**			
*Fixed-effects*			
Intercept	−**0.64 (**−**0.99,** −**0.29)**	−**0.76 (**−**1.20,** −**0.31)**	−**0.80 (**−**1.22,** −**0.39)**
Mass	0.09 (−0.15, 0.33)	−0.07 (−0.26, 0.12)	0.14 (−0.01, 0.29)
Sex			
Male	**0.70 (0.21, 1.21)**	**0.71 (0.08, 1.32)**	0.43 (−0.11, 0.98)
Trial	**0.13 (0.08, 0.18)**	**0.14 (0.06, 0.21)**	**0.31 (0.18, 0.44)**
Time of day^*^	−**0.21 (**−**0.32,** −**0.09)**	−0.05 (−0.16, 0.07)	NA
Sex:trial	−0.06 (−0.14, 0.02)	−0.04 (−0.14, 0.06)	−0.04 (−0.20, 0.12)
*Random-effects*			
Individual ID (intercept)	0.79 (0.61, 1.02)	1.02 (0.81, 1.30)	0.81 (0.58, 1.08)
Trial (slope)	0.09 (0.03, 0.14)	0.16 (0.12, 0.21)	0.17 (0.09, 0.26)
cor(intercept–slope)	−0.17 (−0.59, 0.48)	−**0.76 (**−**0.88,** −**0.58)**	−**0.93 (**−**1.00,** −**0.73)**
**Residual model**			
*Fixed-effects*			
Intercept	−**0.73 (**−**0.86,** −**0.60)**	−**0.59 (**−**0.70,** −**0.46)**	−**0.18 (**−**0.33,** −**0.02)**
Sex			
Male	**0.24 (0.06, 0.42)**	−0.02 (−0.19, 0.15)	−**0.42 (**−**0.64,** −**0.21)**

^*^Time of day was centered (am = −0.5; pm = 0.5) so that positive values represent increased boldness in the afternoons, and vice versa.

### Behavioral predictability

**Figure 3:**
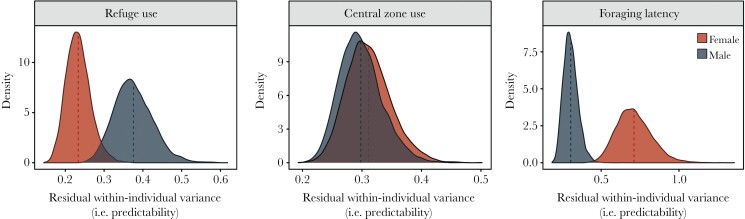
Sex differences in behavioral predictability. Plots represent the posterior probability distributions for residual, within-individual variance (i.e., predictability) in time out of the refuge, time in the central zone, and foraging latency extracted from Bayesian, hierarchical linear mixed-effects models. Distributions are shown for both females (red) and males (blue). The red and blue dotted lines represent posterior means for females and males, respectively.

Like the mean-model, we also found sex differences in behavioral predictability (Table 1). However, this was not consistent across the different assays. In particular, males were less predictable than females in time out of the refuge (Table 1; Figure 3a; Supplementary Figure S1a). However, males were more predictable than females in their foraging latency (Table 1; Figure 3c; Supplementary Figure S1c). In contrast, no sex differences were found in how predictable fish were in their central zone use (Table 1; Figure 3b; Supplementary Figure S1b).

## DISCUSSION

Much research has now found that behavioral predictability can play a role in predator–prey interactions (Jones et al. 2011; Briffa 2013; Chang et al. 2017) and may be underpinned by heritable genetic variation (Martin et al. 2017; Prentice et al. 2020), suggesting that intra-individual behavioral variance may itself be an evolvable trait. Despite this, there is still little research investigating how and why organisms differ in the predictability of their behavior. Here, we provide evidence that variation in behavioral predictability is sex-specific. In particular, we repeatedly measured risk-taking behavior using three different assays in both male and female mosquitofish. We found that individual fish were largely consistent in their mean-level behavior across the three assays, and that male fish were consistently more risk-prone than their female counterparts. Despite the across context consistency of mean-level behavior, both the direction and magnitude of sex differences in behavioral predictability (i.e., residual variance) were assay-specific. These findings highlight that behavioral predictability may be independent of mean-level responses and that males and females may differentially adjust their behavioral consistency in response to subtle changes in environmental context.

In line with predictions and previous research, we found repeatable variation among individuals in their average level of risk-taking behavior (i.e., personality). More specifically, individuals consistently differed from one another in their foraging latency, as well as how much time they spent both out of the refuge and in an exposed central zone. Additionally, we found evidence that this variation was consistent across the three independent assays. Indeed, we found positive among-individual correlations between risk-taking behavior in each context, where individuals that spent more time out of the refuge also spent more time in the exposed central zone, and foraged more rapidly. Much previous research in poecilid fishes has similarly found substantial individual differences in risk-taking behavior (Cote et al. 2010; Polverino et al. 2016; Houslay et al. 2018), and suggested that this variation may be associated with both reproductive success (Wilson et al. 2010; Gasparini et al. 2019; Herdegen-Radwan 2019) and survival (Smith and Blumstein 2010). Together, these results add strong evidence to the growing literature on consistent individual differences in risk-taking behavior, and highlight that refuge-use, thigmotaxis, and foraging latency assays may all provide valid tests of boldness, as they appear to measure the same/similar underlying behavioral trait in fish—at least at the mean-level.

As expected, we also found that males were bolder than females across all three assays. However, we should note that sex differences in foraging latency were only partially supported, with credible intervals overlapping with zero. Nevertheless, these findings are in accordance with previous research in poecilid fish which has also found increased risk-taking behavior in males (Brown et al. 2005; Heinen-Kay et al. 2016; Michelangeli et al. 2020). For example, male Bahamas mosquitofish were significantly bolder than their female counterparts (Heinen-Kay et al. 2016). Further, previous research found that male western mosquitofish (*Gambusia affinis*) were bolder than females (Michelangeli et al. 2020). However, Michelangeli et al. (2020) reported no sex differences in the risk-taking behavior of eastern mosquitofish. Why consistent sex differences in eastern mosquitofish boldness were found in the current study, but not in Michelangeli et al. (2020), is not clear. We surmise that this discrepancy may be partly due to differences in assay setup and/or reproductive season. For example, Michelangeli et al. (2020) tested individual fish for boldness in a standard refuge-emergence assay. Conversely, in the current study, risk-taking behaviors were tested within modified housing tanks, where fish were constantly exposed to olfactory conspecific cues. Male mosquitofish are highly sexually aggressive, with female conspecifics often shoaling to avoid costly male harassment (Pilastro et al. 2003). Thus, female fish may have decreased their risk-taking behavior in the presence of male olfactory cues to avoid male reproductive attempts. Similarly, male fish may have displayed increased boldness in the presence of female cues as they actively sought out mating opportunities. Thus, the presence of intersexual olfactory cues may have contributed to the pronounced sex differences in boldness observed in the current study. Further, while mosquitofish in the present study were collected during the start of the species’ breeding season (i.e., mid-spring), Michelangeli et al. (2020) collected fish during the peak breeding season in mid-summer, when reproductive investment is usually highest (see Pyke 2005 for a review). As previous research has associated boldness with fecundity in eastern mosquitofish (Wilson et al. 2010), variation in reproductive investment across the breeding season may influence sex differences in risk-taking behavior. How the presence of intersexual cues alters the risk-taking behavior of eastern mosquitofish, and whether these effects increase during the species’ breeding season when reproductive activity is highest (Pyke 2005), will be an interesting topic for future research.

We also found strong evidence for sex differences in behavioral predictability. Contrary to our predictions, however, the direction and magnitude of these differences varied across the assays. More specifically, males were less predictable than females in their refuge-use but were more predictable than females in their foraging latency. Meanwhile, there were no sex differences in the predictability of central zone use. This is surprising considering the previously suggested role of increased intra-individual behavioral variation as an adaptive strategy for dealing with predation risk (Briffa 2013; Cornwell et al. 2019; Horváth et al. 2019; but see Urszán et al. 2018), and the sex differences found in both mean-level risk-taking behavior (this study) and vulnerability to predation (Britton and Moser 1982) in mosquitofish. Indeed, one may expect that due to their increased boldness, males would be generally less predictable in their behavior to offset any heighted predation risk. Conversely, one could similarly expect generally decreased behavioral predictability in females as part of a broader risk-management strategy to mitigate their increased vulnerability to predation (Britton and Moser 1982).

In contrast, sex differences in the predictability of risk-taking behavior were highly sensitive to assay conditions. Here, differences between males and females in the costs of risk-taking behavior in each context may party explain the sex differences observed in predictability. For example, due to cost of male sexual harassment and their increased vulnerability to predation (Britton and Moser 1982; Pilastro et al. 2003), female mosquitofish may have consistently sought out shelter, resulting in females being both less risk-prone and more predictable than males during the refuge-use assay. These sex-specific costs may have reversed during the foraging assay. In particular, previous research in Bahamas mosquitofish has suggested that larger female fish face a stronger trade-off between predator avoidance and foraging, relative to smaller male conspecifics (Pärssinen et al. 2021). Females may have, therefore, demonstrated greater within-individual variation (i.e., were less predictable) in their foraging latency as they attempted to balance the costs of perceived predation risk with hunger levels and energy intake over the course of the experiment. Conversely, the lower perceived predation risk of male fish may have enabled them to be both faster and more predictable in their foraging latency. Confirming whether these context-dependent sex differences in behavioral predictability are adaptive and/or due to sex differences in trade-offs between resource acquisition and predation will be an interesting topic for future research.

Further, while previous research investigating sex differences in behavioral predictability has been limited, prior work in the common pill bug and common hermit crab both reported no sex differences in the predictability of risk-taking behavior (White and Briffa 2017; Horváth et al. 2019). Additionally, research into the predictability of behavior in poecilid fish has been mixed, with previous reports in guppies finding sex differences in the predictability of lateralization (McLean and Morrell 2020), but not risk-taking behaviors (Prentice et al. 2020). In light of this, our results suggest that the effects of sex on behavioral predictability are likely to be both species- and context-specific, and highlight that organisms may alter their behavioral consistency in response to subtle changes in environmental conditions. Whether the sex differences in predictability found in the current study are maintained in the presence of acute predation risk—when the cost of being bold and more predictable may be highest—is not known. Future research testing the risk-taking behavior of fish both in the presence and absence of predation threat will be needed to understand how males and females may differentially adjust the predictability of their behavior in response to increased risk.

The underlying mechanisms that promote these sex differences in predictability are also not clear. In this regard, previous research has associated variation in behavioral predictability with key energetic traits (Velasque and Briffa 2016; Biro et al. 2018). Indeed, prior analysis of voluntary wheel-running behavior in selectively bred laboratory mice found that lines with greater aerobic scope (i.e., difference between maximum and minimum metabolic rate) were more unpredictable in their behavior (Biro et al. 2018). Here it is thought that individuals with greater aerobic scope may have an increased capacity to express behavioral variation, and thus will be more unpredictable in their behavior (Biro et al. 2018). While previous research in eastern mosquitofish reported increased maximum metabolic rates in males when compared to females, there were no sex differences in aerobic scope (Srean et al. 2017). Whether differences between males and females in energetic traits vary with risk level and explain context-specific sex differences in behavioral predictability will be key to understanding the proximate mechanisms that drive variation in the consistency of behavioral traits.

In summary, we found strong evidence that sex differences in the predictability of risk-taking behavior were assay-specific. This is despite finding that individuals were generally consistent in their mean-level behavior across the assays, and that males and females consistently differed in their mean-level risk-taking behavior. These results also highlight that while the three assays used here to investigate risk-taking behavior seem to measure the same and/or similar underlying behavioral trait at the mean-level, there are subtle differences in how fish modified their behavior within each context at the residual level. We suggest that future studies taking repeated measures of risk-taking behavior and energetic traits—in both the presence and absence of predation threat—will provide insight into relationships between context-specific variation in behavioral predictability and organismal physiology. Such research will be needed to determine both the proximate and ultimate mechanisms that determine sex differences in behavioral consistency.

## Supplementary Material

arac105_suppl_Supplementary_Material

## Notes

We would like to thank Rachel Mason, Jack Orford, and Marcus Michelangeli for assistance with fish husbandry and filming behavioral trials. We would also like to thank Lesley Alton, Peter Biro, Daniel Bolnick, Mark Briffa, Rebecca Fuller, and two anonymous reviewers for advice which greatly improved this manuscript.
