# Supplementary material for: Sex differences in the predictability of risk-taking behavior
Source: Behav Ecol. 2022 Dec 13;34(1):108–16. doi: 10.1093/beheco/arac105 (PMC9918862; doi:10.1093/beheco/arac105)
Supplement: arac105_suppl_Supplementary_Material [file arac105_suppl_supplementary_material.pdf]

Sex differences in the predictability of risk-taking behaviour

Electronic supplementary material

METHODS

Behavioural experiments and video processing

Behavioural video files were processed using previously established methods (Douek et al. 2021; Henry et al. 2019). Video files were post-processed using DaVinci Resolve 15 (BlackMagic Design, Australia) video editing software. Post-processing involved sharpening, baseline contrast correction, and background subtraction techniques. Corrected files were then analysed using EthoVision XT v.15 tracking software (Noldus Information Technology, Wageningen, the Netherlands). Digital video-based tracking of animal behaviour was based on a reconstruction of movement pattern analysis in a grid of pixels on individual frames of the video files. Software algorithms analysed each frame of the video file to distinguish the tracked animals from the background. This was performed based on semi-automated adjustment of threshold of pixel intensity and colour saturation values. Each detected animal was automatically assigned a mathematical centre of gravity (centroid) derived from the average surface area. Automatic frame-by-frame tracking produced time-stamped *x,y* coordinate pairs assigned to centroids of detected objects and provided a foundation for the behavioural parameters (i.e. time spent in refuge and central zone) to be calculated for each arena. Zones of interest were defined in order to calculate the edge-preference index of the fish.

During the thigmotaxis assay, we identified two trials in which the program failed to track the fish. Video recordings for these trials were therefore manually watched by an experimental observer (JAB). We confirmed that the fish during these trials remained up against the walls of the tank throughout the entire duration of the trial and, therefore, never entered the exposed central zone. These fish were given scores of 0 for total time spent within the central zone. Further, one individual did not complete the whole experiment and, therefore, is missing data for 2 refuge use and thigmotaxis trials each, and 1 foraging trial. However, the data from the initial behavioural observations from this individual were retained in the analysis.

Statistical analysis

We used a multivariate model to estimate correlations among each of our measures of risk-taking behaviour across individuals. However, there were some missing values in the dataset. These were due to some individuals not completing the full experiment (see supplementary materials – Behavioural experiments), and the increased number of repeated measures for refuge use and thigmotaxis trials, when compared to foraging trials. We, therefore, used multiple imputation (*mi* function; *brms* package; Bürkner 2017) during model fitting to account for missing values in each response variable as suggested by Nakagawa and Freckleton (2008, 2011) and used in Mitchell et al. (2020). Multiple imputation infers missing values from the posterior predictive distribution of the relevant response variable (the default case is to delete rows with missing data). This allowed us to retain the maximum amount of data in the model to estimate among-individual correlations between all three measures of risk-taking behaviour.

RESULTS

Table S1. Schedule for the three assays (i.e. refuge-use, thigmotaxis, and foraging) during the experimental period. Feeding during routine husbandry and water changes are also displayed. This schedule was the same for all individuals in the experiment.

	Experimental day						
**Week**	**Day 1**	**Day 2**	**Day 3**	**Day 4**	**Day 5**	**Day 6**	**Day 7**
1	**AM:** Refuge	**AM:** Thigmotaxis	**AM:** Refuge	**AM:** Thigmotaxis	**AM:** Refuge	**AM:** —	**AM:** —
	**PM:** Refuge	**PM:** Thigmotaxis, Foraging trial	**PM:** Refuge, Fed (husbandry)	**PM:** Thigmotaxis, Foraging trial	**PM:** Refuge, Foraging trial	**PM:** Fed (husbandry), Water change	**PM:** —
2	**AM:** Thigmotaxis	**AM:** Refuge	**AM:** Thigmotaxis	Fed (husbandry)	Fed (husbandry)	Fed (husbandry)	End
	**PM:** Thigmotaxis, Foraging trial	**PM:** Refuge, Foraging trial	**PM:** Thigmotaxis		Body mass measurements	Body mass measurements, Water change	

Table S2. Model output from Bayesian, hierarchical generalized linear mixed-effects models, excluding the individual for which mass was estimated from sex-specific means. Estimates with 95% credible intervals are presented for each separate measure of risk-taking behaviour. Bold text indicates fixed-effects estimates and intercept-slope correlations which were different from zero. Note: random effects are presented in standard deviation (sd) units and the residual model is presented on the log scale.

	Time out of refuge	Time in central zone	Foraging latency (inverted)
**Mean model**			
Fixed-effects			
Intercept	**–0.67 (–1.02, –0.31)**	**–0.83 (–1.26, –0.39)**	**–0.77 (–1.18, –0.35)**
Mass	0.09 (–0.16, 0.33)	–0.08 (–0.27, 0.12)	0.14 (–0.00, 0.28)
Sex			
Male	**0.73 (0.22, 1.23)**	**0.77 (0.17, 1.36)**	0.40 (–0.18, 0.96)
Trial	**0.13 (0.08, 0.19)**	**0.14 (0.07, 0.22)**	**0.33 (0.20, 0.45)**
Time of day*	**–0.20 (–0.32, –0.08)**	–0.04 (–0.15, 0.07)	NA
Sex:trial	–0.06 (–0.14, 0.02)	–0.05 (–0.15, 0.06)	–0.05 (–0.21, 0.11)
Random-effects			
sd(intercept.individualID)	0.80 (0.61, 1.03)	0.99 (0.79, 1.26)	0.83 (0.61, 1.10)
sd(slope.trial)	0.09 (0.03, 0.14)	0.16 (0.12, 0.21)	0.18 (0.11, 0.27)
cor(intercept–slope)	–0.17 (–0.60, 0.44)	**–0.75 (–0.87, –0.57)**	**–0.94 (–1.00, –0.80)**
**Residual model**			
Fixed-effects			
Intercept	**–0.72 (–0.85, –0.59)**	**–0.62 (–0.74, –0.50)**	**–0.29 (–0.45, –0.14)**
Sex			
Male	**0.23 (0.05, 0.40)**	0.01 (–0.15, 0.18)	**–0.31 (–0.53, –0.09)**

Note: time of day was centred (AM = –0.5; PM = 0.5) so that positive values represent increased boldness in the afternoons, and vice versa.

Table S3. Output from Bayesian multivariate linear mixed-effects model using multiple imputation to account for missing values in the data. Estimates with 95% credible intervals (CrI) are presented for each separate measure of risk-taking behaviour. Bold text indicates estimates which were significantly different from 0. Note: random effects are presented in standard deviation (i.e. sd) units.

	**Time out of Refuge**	**Time in central zone**	**Foraging latency**
**Mean model**			
Fixed-effects			
Intercept	**–0.30 (–0.54, –0.05)**	**–0.40 (–0.69, –0.11)**	**–0.58 (–0.84, –0.33)**
Mass	0.08 (–0.14, 0.31)	–0.07 (–0.27, 0.12)	0.09 (–0.10, 0.28)
Sex*	**0.70 (0.24, 1.18)**	**0.69 (0.13, 1.26)**	0.42 (–0.10, 0.96)
Trial	**0.10 (0.06, 0.15)**	**0.12 (0.06, 0.17)**	**0.29 (0.21, 0.37)**
Time of day*	**–0.20 (–0.32, –0.09)**	–0.05 (–0.16, 0.06)	NA
Sex:trial	–0.06 (–0.14, 0.03)	–0.04 (–0.14, 0.06)	–0.03 (–0.19, 0.13)
Random-effects			
sd(intercept.individualID)	0.76 (0.59, 0.96)	0.96 (0.77, 1.18)	0.74 (0.51, 1.00)
sd(slope.trial)	0.10 (0.06, 0.14)	0.16 (0.12, 0.20)	0.16 (0.06, 0.27)
cor(intercept–slope)	–0.16 (–0.53, 0.26)	**–0.72 (–0.85, –0.53)**	**–0.65 (–0.90, –0.20)**
Residuals	0.55 (0.50, 0.59)	0.55 (0.51, 0.60)	0.69 (0.62, 0.78)

* Sex and time of day were coded as mean-centred covariates (i.e. female = –0.5; male = 0.5: AM = –0.5; PM = 0.5) so that positive values represent an increase in boldness in males and an increase in boldness in the afternoon, respectively.

Table S4. Estimates (± 95% CrI's) of conditional repeatability at the intercept (i.e. trial 1), trial 4, and trial 7 for time out of the refuge, time in the central zone, and foraging latency. Note: repeatability at trial 7 is not included for foraging latency as only 5 foraging trials were conducted.

Behavioural traits	Trial 1 (intercept)	Trial 4	Trial 7
Time out of the refuge	0.655 (0.536, 0.771)	0.660 (0.551, 0.764)	0.717 (0.604, 0.818)
Time in the central zone	0.748 (0.661, 0.834)	0.613 (0.510, 0.727)	0.618 (0.495, 0.741)
Foraging latency	0.523 (0.331, 0.705)	0.381 (0.210, 0.556)	NA

Table S5. Plasticity syndromes. Estimates (± 95% CrI's) for correlation among slopes (i.e. trial number) extracted from the Bayesian multivariate model using multiple imputation to account for missing values in the data. Bold text indicates correlations which were significantly different from 0.

Correlations	Estimate (± 95% CrI's)
Time out of the refuge (slope), Foraging latency (slope)	0.128 (–0.414, 0.629)
Time out of the refuge (slope), Time in the central zone (slope)	**0.613 (0.251, 0.878)**
Time in the central zone (slope), Foraging latency (slope)	0.237 (–0.229, 0.668)

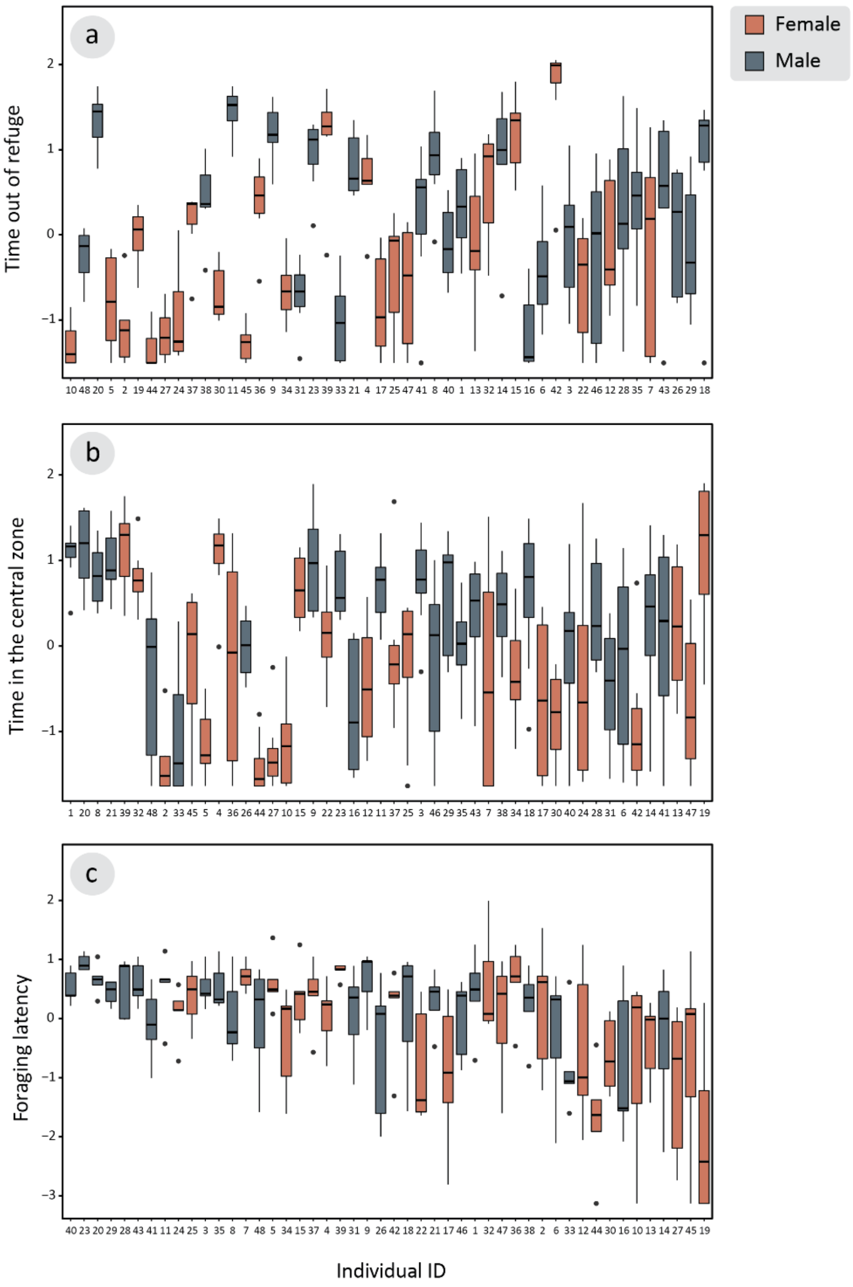

Figure S1. Individual boxplots for (a) time out of the refuge, (b) time in the central zone, and (c) foraging latency for both females (red) and males (blue). Boxplots are arranged from left to right by the absolute values of each individual's residual variance (i.e. predictability) extracted from the univariate Bayesian, hierarchical linear mixed-effects models, whereby those on the left were the most predictable, and *vice versa* for individuals on the right. Behavioural scores are presented in transformed (see statistical analysis methods) and standardised units.

REFERENCES

Bürkner PC. 2017. brms: An R package for Bayesian multilevel models using Stan. Journal of Statistical Software. 80:1–28.

Douek AM, Amiri Khabooshan M, Henry J, Stamatis S-A, Kreuder F, Ramm G, et al. 2021. An engineered sgsh mutant zebrafish recapitulates molecular and behavioural pathobiology of sanfilippo syndrome A/MPS IIIA. International Journal of Molecular Sciences. 22:5948.

Henry J, Rodriguez A, Wlodkowic D. 2019. Impact of digital video analytics on accuracy of chemobehavioural phenotyping in aquatic toxicology. PeerJ. 7:e7367.

Mitchell DJ, Beckmann C, Biro PA. 2020. Predation as a driver of behavioural variation and trait integration: effects on personality, plasticity, and predictability. EcoEvoRxiv.

Nakagawa S, Freckleton RP. 2008. Missing inaction: the dangers of ignoring missing data. Trends in Ecology and Evolution. 23:592–596.

Nakagawa S, Freckleton RP. 2011. Model averaging, missing data and multiple imputation: A case study for behavioural ecology. Behavioral Ecology and Sociobiology. 65:103–116.
